# Oral health seeking behaviors of adults in Nebbi District, Uganda: a community-based survey

**DOI:** 10.1186/s12903-021-01824-5

**Published:** 2021-09-17

**Authors:** Juliet Ocwia, Ronald Olum, Pamela Atim, Florence Laker, Jerom Okot, Senai Goitom Sereke, Joseph Baruch Baluku, Sarah Kiguli, Felix Bongomin

**Affiliations:** 1 Faculty of Medicine, Gulu University, Gulu, Uganda; 2 School of Medicine, College of Health Science, Makerere University, Kampala, Uganda; 3 Kiruddu National Referral Hospital, Kampala, Uganda

**Keywords:** Oral health, Dental caries, Utilization, Associated factors, Knowledge, Nebbi, Uganda

## Abstract

**Background:**

Dental health is often neglected by the majority of the population and has contributed to the global burden of oral diseases. We assessed awareness, utilization and barriers to seeking oral health care among adults in Nebbi District, Uganda.

**Methods:**

A community-based, cross sectional study was conducted in the central division, Nebbi District in Uganda among adults between the age of 18 years or older. An interviewer-administered, semi-structured questionnaire was used for data collection on socio-demographic characteristics, oral health awareness, oral health utilization, associated factors and barriers.

**Results:**

A total of 400 adults with a median age of 32 years (interquartile range 24–43) years were enrolled. More than half (57.5%, n = 230) of the participants were female. Participants identified smoking (42.8%, n = 171) and consumption of sugary foodstuffs (29.0%, n = 116) as risk factors for oral disease. Not brushing was also identified by 260 participants (65.0%) as the cause of tooth decay and 95.8% (n = 383) believed brushing one’s teeth could prevent tooth decay. Of the 51.5% (n = 206) who had experienced a toothache or discomfort 12 months prior to the study but only about half (52%, n = 106) had sought healthcare from a dental clinic or facility. About 89.5% (n = 94) of the participants were able to see a dentist during their last visits. Dental carries (76.6%, n = 70) and gum bleeding (14.9%, n = 14) were the most frequent reasons for visiting a dental clinic, and 73.7% (n = 70) had their tooth extracted. Barriers to seeking oral healthcare were cost of treatment (47.5%, n = 190), and long waiting time (18.5%, n = 74). The odds of seeking oral healthcare was 2.8-fold higher in participants who were being married (Odds ratio (OR): 2.8, 95% CI 1.3–6.3, *p* = 0.011) and 3.5-fold higher among housewives (COR: 3.5, 95% CI 1.1–11.4, *p* = 0.040).

**Conclusion:**

About half of the participants had sought healthcare following a dental condition. Cost of treatment seems to be an important factor affecting utilization of oral health services. Optimization of costs, and creating awareness regarding benefits of utilizing preventive dental services are recommended.

**Supplementary Information:**

The online version contains supplementary material available at 10.1186/s12903-021-01824-5.

## Background

Oral diseases represent a major public health challenge globally, and yet remain neglected. It is estimated that oral diseases affect over 3.5 billion people worldwide, with dental caries of permanent teeth being the most common condition [1, 2]. Globally, it is estimated that 2.3 billion people suffer from caries of permanent teeth and more than 530 million children suffer from caries of primary teeth [2].

The burden of oral diseases is more severe in poor and disadvantaged population groups. A systematic review has suggested a 20.8% increase in the global burden of oral conditions in the past decades, and this has been attributed to population growth and aging [2]. HIV/AIDS is also an important risk factor for oral diseases, especially in sub-Saharan Africa which has the highest burden of HIV [3].

In Uganda, there is inadequate data on the oral health. A community-based oral health survey reported that just over half of adults in communities in Uganda had at least one oral health concern 6 months prior to the survey. Of these, only about one-third received the appropriate treatment [4]. In this survey, tooth decay was found to be the most common oral health concern and several underlying risk factors such as oral hygiene, health seeking behaviors and health systems factors were identified [4].

Despite the high levels of oral diseases in Uganda [5], utilization of oral health services is still very low as people visit dental clinics for pain relief or in need of emergency services [6]. Utilization of oral health services and its barriers become important parameters in oral health planning [6]. This is because they provide useful information and guidance to health planners and policy makers to help in developing appropriate structures and appropriate resource allocation [6].

The purpose of this study was to assess the awareness, utilization and barriers to seeking oral health among adults in Nebbi District, Uganda.

## Methods

### Study design

This was a community-based, cross sectional study with a quantitative approach. The study was conducted between October and December 2020.

### Study setting

The study was conducted in Nebbi district, a rural district in Uganda, located in West-Nile sub-region, Northern Uganda. Nebbi has a municipal council which has three divisions namely Central, Thatha and Abindu division. This study was conducted in the central division. Nebbi District has a population of 282,600 (Male: 49%; Female: 51%), 145,400 female, 137,200 males (UBOS Population projection 2020). Nebbi district has two public health center (HC) level four (HC IV) and two general hospitals which provide dental health services [7]. In Uganda, an HC IV offers a comprehensive medical, surgical and maternity services including dentistry. The unit is headed by a medical doctor. Dental services at HCIV is offered by a dental public health practitioners and nurses trained in dental health care.

### Study population

The target population comprised of community members living in the rural areas of Central Division, Nebbi District, Uganda.

### Sampling method

We used simple random sampling to select households. Everyone in the selected household was eligible for the study as long as he or she was within the age limits and was willing to provide informed consent.

### Sample size calculation

The sample size was calculated using Kish-Leslie formula (1965) as summarized below:

$${\text{N}} = \frac{{Z^{2} p\left( {1 - p} \right)}}{{d^{2} }}$$

where N = Sample size required. Z = standard normal distribution abscissa corresponding to 95% confidence interval (1.96). P = Proportion of persons underutilizing oral health services. An estimated value of 50% was used (0.5) since the proportion of persons underutilizing oral health care is not known. D = Desired level of precision/marginal error (0.05)

$${\text{N}} = \frac{{Z^{2} p\left( {1 - p} \right)}}{{d^{2} }}$$

N = (1.96^2^ × 0.5(1–0.5))/0.05^2^. N = 384. Non-response of 10% was 38 people. Hence the sample size was 422.

### Selection criteria

We included all adults aged 18 to 65 years old who consented to participate were included in the study. Persons who were too sick and mentally ill to participate were not included in the study.

### Data collection

A semi-structured questionnaire (Additional file 1) was used to collect quantitative data on: demographics (age, sex, address, religion, occupation, and marital status), source of income, estimated monthly income, household population, and distance to nearest health facility, awareness on oral health, oral health seeking among individuals with an oral complaint and barriers to seeking oral health services. The questionnaire was pre-tested in another division to ensure its reliability and validity in assessing the study variables. Cronbach alpha score was 0.80 for all questions showing a very high internal validity. During the study, the questionnaires was assessed for completeness and kept safely awaiting data analysis. We collected data using an interviewer -administered semi structured questionnaire. All interviewers were trained nurses who underwent a two-day training on the study and the data collection tool. All interviewers participated in the pilot study and pre-testing of the study tool.

### Data analysis

Data was analyzed using STATA version 16.0 Categorical data was summarized into frequencies and percentages and numerical data as means and standard deviations or medians and interquartile ranges (IQR) as appropriate. Chi-square tests were performed to look for association between categorical independent variables and the dependent variables and student t-test or Mann–Whitney U tests were used to assess for association between numerical independent variables and the dependent. Multivariable logistic regression was performed for all variables with a *p* < 0.2 at bivariate analysis to assess for factors associated with seeking of dental services. A *p* < 0.05 was considered statistically significant.

### Ethical considerations

The research proposal was approved by the Gulu University Research & Ethics committee. All participants provided informed written consents. All methods were performed in accordance with the Declaration of Helsinki.

## Results

### Socio-demographic data of the participants

Of the anticipated 422 participants, a total of 400 adults (response rate 95%), with a median age of 32 years (IQR: 24 to 43 years) participated in the study. More than half were female (57.5%, n = 230), aged 18 to 35 years (58.5%, n = 234) and had attained primary education (56.0%, n = 224). About two-thirds were married (68.5%) and were farm laborers (62.2%). The main source of income was farming (45%), with an estimated median monthly income of 50,000 UGX (IQR: 20,000 UGX to 100,000 UGX; 13.5 US dollars (USD), IQR-5.5 USD to 27 USD). Table 1 summarizes the social and demographic characteristics of the participants.

**Table 1:** Characteristics of participants

Variable	Frequency (*N* = 400)	Percent (%)
*Age in years (median, IQR)*	32	24–43
18–35 years	234	58.5
36–60 years	143	35.8
> 60 years	23	5.8
*Education level*		
No formal education	62	15.5
Primary	224	56.0
Secondary	90	22.5
Tertiary/university	24	6.0
*Occupation (n* *=* *397)*		
Farm labourers	247	62.2
Business	95	23.9
Housewife	34	8.6
Civil servant	21	5.3
*Marital status*		
Married	274	68.5
Single	76	19.0
Divorced/separated	37	9.3
Widow/widower	13	3.3
*Source of income*		
Farming	179	45.0
Business	148	37.2
Support from friends or family	52	13.1
Formal employment	19	4.8
Number of people in homestead (n = 386), median, range	6	4–8

### Knowledge and practices towards oral hygiene

Table 2 summarizes the knowledge and practices of adults towards oral hygiene. The most frequently identified risk factors for oral disease were smoking (42.8%, n = 171) and consumption of sugary foodstuffs (29.0%, n = 116). Some 23.3% (n = 93) did not know any risk factors. Not brushing was also identified by 260 participants (65.0%) as the cause of tooth decay and the greatest majority believed brushing one’s teeth could prevent tooth decay (95.8%, n = 383). Some 79 adults (19.8%) used sticks for brushing and 6 participants each using either soap or sand. 56.5% (n = 226) reported brushing their teeth at least twice a day.

**Table 2:** Knowledge and practices towards oral hygiene among adults

Question	Frequency (N = 400)	Percent (%)
*Risk factors for oral disease*		
Smoking	171	42.8
Consumption of sugary foodstuffs	116	29.0
I don't know	93	23.3
Alcohol consumption	17	4.3
Sweet drinks at bedtime	3	0.8
*Cause of tooth decay*		
Not brushing	260	65.0
I don't know	69	17.3
Consumption of sugary foodstuffs	61	15.3
Hereditary	10	2.5
*How to keep your teeth clean*		
Brushing the teeth	383	95.8
I don't know	10	2.5
Avoid eating sweet foodstuffs	4	1.0
Avoid sweet drinks at bedtime	2	0.5
Avoid smoking	1	0.3
*What do you use for cleaning your teeth?*		
Toothbrush	315	78.8
Sticks	79	19.8
Sand	3	0.8
Soap	3	0.8
*How often do you brush in a day?*		
Once	174	43.5
Twice	156	39.0
Thrice	70	17.5
*Duration taken while brushing the teeth (n* *=* *398)*		
I don't know	264	66.3
2 min	69	17.3
1 min	49	12.3
20 s	16	4.0

### Utilization of oral health services

Up to 51.5% (n = 206) had experienced a toothache or discomfort 12 months prior to the study (Table 3). Of these, only about half (52%, n = 106) had sought healthcare from a dental clinic or facility. The major reason for seeking care was toothache (86.7%, n = 91). Eighty-nine percent (n = 94) were able to see a dentist during their last visits. Dental caries (76.6%) and gum bleeding (14.9%) were the most frequent treated conditions, and the majority were treated by tooth extraction (73.7%, n = 70). Most participants reported that the dentist was friendly during their care and treatment (80.2%, n = 77). Marital status was significantly associated with utilization of oral health services (*p* = 0.013). The odds of seeking oral health services was 3.5-fold higher among housewives (COR: 3.5, 95% CI 1.1–11.4, *p* = 0.040) and 2.8-fold higher among married participants (COR: 2.8, 95% CI 1.3–6.3, *p* = 0.011). However, multivariable logistic regression did not reveal statistically significance.

**Table 3:** Utilization of oral health services among adults

Variable	Frequency	%
*Toothache or oral discomfort in the past 12 months (n* *=* *400)*		
Yes	206	51.5
No	194	48.5
*Level of utilization of oral health services following an oral condition in the past 12 months (n*** = **204)		
No	98	48.0
Yes	106	52.0
*The main reason for your last visit to dental clinic (n* = *105)*		
Painful tooth	91	86.7
Regular check-up	10	9.5
Follow-up	2	1.9
Sensitivity of the teeth	2	1.9
*Were you able to see the dentist? (n* = *105)*		
No	11	10.5
Yes	94	89.5
*What conditions were you treated for? (n*** = ***94)*		
Caries	72	76.6
Bleeding gum	14	14.9
Dental abscess	3	3.2
Fractured tooth	3	3.2
Sensitivity of the teeth	2	2.1
*Types of treatment received*		
Tooth extraction	70	73.7
Medication	18	18.9
Cleaning	4	4.2
Filling	2	2.1
Oral education	1	1.1
*Means to reach the health facility for oral care? (n* *=* *96)*		
Walking	72	75.0
Boda-Boda*	23	24.0
Public means(vehicle)	1	1.0
*How long did it take you to receive help? (n* *=* *96)*		
3 h	34	35.4
1 h	22	22.9
2 h	22	22.9
30 min	18	18.8
*What was the dentist’s response towards you? (n* *=* *96)*		
Friendly	77	80.2
Busy	10	10.4
Rude	9	9.4

*Boda-Boda is a form of bicycle and motorcycle taxis commonly used in Uganda. Transport fares are charged according to the distance covered

### Barriers to seeking healthcare for dental conditions

**Fig. 1:**
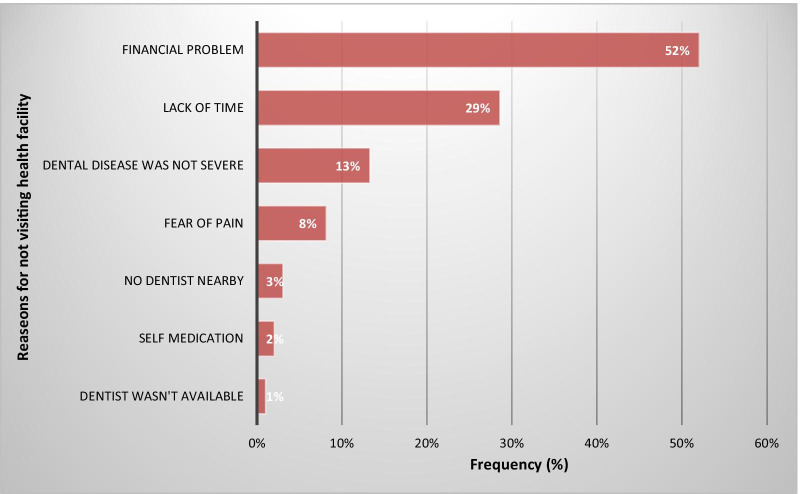
Reasons for not visiting the health facility during the last episode of tooth discomfort (n = 98)

Financial problems, lack of time and non-severe dental diseases were the most common reasons (Fig. 1). The five most frequent reasons for barriers to seeking dental healthcare were, (I) cost of treatment (47.5%, n = 190), (II) long waiting time (18.5%, n = 74), (III) distance to the facility (9.8%, n = 39), (IV) fear of pain (8.5%, n = 34) and (V) attitudes of the dentists (3.8%, n = 15).

## Discussion

This study sought out to determine the practices, utilization, and barriers to oral health services in a rural district in Northwestern Uganda. The results of our study show that half of the participants had experienced a toothache or discomfort 12 months prior to the study. However, only about half of these had sought healthcare from a dental clinic or facility. The most reported reason for not seeking dental care is the widely held perception that one needs to visit dentist only when there are symptoms such as pain and emergency. Many studies show that one key reason for this is the belief that oral diseases are not serious or life threatening. Studies done in India show that about between 62.5% and 70% of the participants believed that unless there is pain, there is no need to visit a dentist [8, 9].

The major reason for seeking oral healthcare was toothache. This is supported by study done in India where reported reasons for visiting dentist in the past were related to having oral symptoms or pain [10, 11]. In other studies, done in Tanzania, dental pain and discomfort were cited as common reasons for seeking dental care [12, 13]. Kadarulu and colleagues also found in their study that the majority of the dental visits were for pain relief [14]. Visiting the dentist for regular check-up as a preventive measure of common oral diseases, was not well known. In this study, only 9.5% of the participants mentioned it as a measure of oral disease prevention meaning early identification and prevention of oral diseases are always made difficult since most people came at later stages of the disease leaving them with no other options of treatment other than tooth extraction, which is similar to a study conducted in India where the people interviewed said they had never visited a dentist for routine oral examination [15].

The results of this study showed that most participants had good knowledge about the risk factors for oral disease. Majority of them identified smoking (42.8%) and consumption of sugary foodstuffs (29.0%) were risk factors for oral disease. Some 23.3% did not know any risk factors. Risk factors for oral diseases include unhealthy eating habits or diet, tobacco use, harmful alcohol use, and poor oral hygiene [16]. In our study, knowledge on prevention of oral diseases varied as the greatest majority of the participants knew that brushing teeth thoroughly prevents cavities, but only a small percentage knew that one form of prevention of dental caries is to avoid eating sugary foods. The international studies have showed that patients with generally higher levels of education than our study participants (for example university students in Ethiopia and teachers and students in India) had greater knowledge of preventive measure of gum diseases and caries [17, 18]. Education level was however not significant in our study.

In this study, 9.8% of the participants expressed that distance to the facility hinders them from accessing oral health service because they have to walk long distances in order seek of oral care. Similar studies also show that people in rural areas often have greater distances to travel to reach health care services where the availability of transport is limited and costs higher [19, 20]. There are noted differences between urban and rural areas and proximity to oral health services.

Our study has important limitations. The sample size is small and mainly rural. Therefore, our study may not be generalized to the entire Ugandan population. However, we provide an important baseline epidemiological study to inform future public health surveillance and interventions.

## Conclusion

In conclusion, whereas most of the participants had good awareness on oral health, only about half of them sought oral health services for an oral health condition in the past twelve months. Cost of treatment, long waiting time and distance to the health facility were frequently reported barriers.

## Supplementary Information

**Additional file 1**. A semi-structured questionnaire.

## Data Availability

The datasets used and/or analyzed during the current study are available from the corresponding author on reasonable request.
