# Supplementary material for: Oral health seeking behaviors of adults in Nebbi District, Uganda: a community-based survey
Source: BMC Oral Health. 2021 Sep 17;21:453. doi: 10.1186/s12903-021-01824-5 (PMC8447567; doi:10.1186/s12903-021-01824-5)
Supplement: Supplementary file 1 — Additional file 1. A semi-structured questionnaire. [file 12903_2021_1824_MOESM1_ESM.docx]

## QUESTIONAIRE

**Questionnaire Number [ | | ]**

This questionnaire is designed to capture information on utilization of oral health services and associated factors among adults. The questionnaire must be administered to only persons who have consented to participate in the study

**SECTION A: DEMOGRAPHIC INFORMATION: (Tick the correct answer)**

**Gender:** Male Female

1**. Age in years: __________________**

2. **Address:** ______________________

**3. Education level**

Informal Primary Secondary Tertiary /university

**4. Religion**

Catholic Protestant Islam Others_______________

**5. Occupation:**

Civil Servant Peasant Housewife Business

**6. Marital status:**

Single Married Divorced/ Separated Widow/Widower

**7. Source of income**

Formal Employment Business Support from friends or family

Others (specify) ----------------------------------

8. Estimated monthly income----------------------------------UGX

9. How many of you live in the same house? -----------------------------------

**SECTION B: LEVEL OF UTILIZATION OF ORAL HEALTH SERVICES**

1. Have you had any toothache or discomfort in the past 12 months?

Yes No

1. If yes, when was the last time you made the visit?

Six Months More than six months but less than a year

More than one year but less than two years never have been

3. What was the main reason for your last visit to dental clinic?

Regular check-up Painful tooth Follow- up

Others (specify) __________________

4. Were you able to see the dentist?

Yes No

5.  If no, what was the reason?

No dental diseases Dental disease was not severe

No need to cure primary teeth Economic issue

Inconvenience No time

Fear of pain No dentists nearby

Fear of infectious diseases No reliable dentists

Difficulty of registration other reasons____________________________

6. What conditions were you treated for?

Caries Bleeding gum others (specify) ________________

7. What types of treatment were you given?

Tooth extraction Filling Cleaning others (specify) ________________

8. How do you think of the following points of view? **(Choose only one answer for every sub- question)**

|  | 1 | 2 | 8 | 9 |
| --- | --- | --- | --- | --- |
|  | agree | disagree | do not care | unknown |
| 1. Oral health is very important to our life. | ⬜ | ⬜ | ⬜ | ⬜ |
| 1. Regular oral examination is essential | ⬜ | ⬜ | ⬜ | ⬜ |
| 1. It is your responsibility to prevent tooth disease | ⬜ | ⬜ | ⬜ | ⬜ |
| 1. It's important to protect your teeth | ⬜ | ⬜ | ⬜ | ⬜ |
| 1. Poor conditions of your teeth can affect other organs like the heart | ⬜ | ⬜ | ⬜ | ⬜ |

**SECTION C- FACTORS AFFECTING UTILIZATION**

9. What things do you think make people not seek for dental care at health facilities?

Long waiting time Distance to the facility Cost of treatment

Attitude of the dentist

Others (specify) -------------------------------------------------------------------------------------

10. How far is the health facility from your home?

1/2 km 1 km 2 km 3km

Others (specify) ----------------------------------------------------------------------------------------

11. What means do you use to reach the health facility for oral care?

Walking Boda-boda Public means (vehicle

12. How long did it take you to receive help?

More than 30 minutes 1 hour 2 hours 3 hours

13. What was his response towards you?

Rude Friendly Busy

14. What do you think are the risk factors for oral disease?

Alcohol consumption Smoking Sweet drinks at bedtime

I don’t know Consumption of sugary foodstuffs

15. What do you think is the cause of tooth decay?

Not brushing Consumption of sugary foodstuffs

Hereditary I don’t know

16. What can you do to keep your teeth clean?

------------------------------------------------------------------------------------------------------------------------------------------------------------------------------------------------------------------------------------------------------------------------------------------------------------------------------------------------

17. What do you use for cleaning your teeth?

Toothbrush Sticks Sand Soap

Others (specify) ----------------------------------------------------------------------------------------

18. How often do you brush in a day? ------------------------------------------------------------------

19. How long do you take brushing your teeth?

20 Seconds 1 minute T2 2 minutes I d I don’t know
